# Exosomal microRNA-342-5p secreted from adipose-derived mesenchymal stem cells mitigates acute kidney injury in sepsis mice by inhibiting TLR9

**DOI:** 10.1186/s12575-023-00198-y

**Published:** 2023-04-21

**Authors:** Wei Liu, Chenghuan Hu, Buyao Zhang, Mingxia Li, Fuxing Deng, Shuangping Zhao

**Affiliations:** 1 Department of Critical Care Medicine, Xiangya Hospital of Central South University, Changsha, Hunan People’s Republic of China; 2 Hunan Provincial Clinical Research Center for Critical Care Medicine, Xiangya Hospital of Central South University, Changsha, Hunan People’s Republic of China; 3 National Clinical Research Center for Geriatric Disorders, Xiangya Hospital of Central South University, Changsha, Hunan People’s Republic of China

**Keywords:** Acute kidney injury, Adipose-derived mesenchymal stem cells, Exosome, miR-342-5p, TLR9, Autophagy

## Abstract

**Background:**

Sepsis-related acute kidney injury (AKI) is an inflammatory disease associated with extremely high mortality and health burden. This study explored the possibility of exosomes secreted by adipose-derived mesenchymal stem cells (AMSCs) serving as a carrier for microRNA (miR)-342-5p to alleviate sepsis-related AKI and investigated the possible mechanism.

**Methods:**

Serum was obtained from 30 patients with sepsis-associated AKI and 30 healthy volunteers for the measurement of miR-342-5p, blood urea nitrogen (BUN), and serum creatinine (SCr) levels. For in vitro experiments, AMSCs were transfected with LV-miR-342-5p or LV-miR-67 to acquire miR-342-5p-modified AMSCs and miR-67-modified AMSCs, from which the exosomes (AMSC-Exo-342 and AMSC-Exo-67) were isolated. The human renal proximal tubular epithelial cell line HK-2 was induced by lipopolysaccharide (LPS) to construct a cellular model of sepsis. The expression of Toll-like receptor 9 (TLR9) was also detected in AKI cells and mouse models. The interaction between miR-342-5p and TLR9 was predicted by dual luciferase reporter gene assay.

**Results:**

Detection on clinical serum samples showed that BUN, SCr, and TLR9 were elevated and miR-342-5p level was suppressed in the serum of patients with sepsis-associated AKI. Transfection with LV-miR-342-5p reinforced miR-342-5p expression in AMSCs and AMSC-secreted exosomes. miR-342-5p negatively targeted TLR9. LPS treatment enhanced TLR9 expression, reduced miR-342-5p levels, suppressed autophagy, and increased inflammation in HK-2 cells, while the opposite trends were observed in LPS-induced HK-2 cells exposed to AMSC-Exo-342, Rapa, miR-342-5p mimic, or si-TLR9. Additionally, the effects of AMSC-Exo-342 on autophagy and inflammation in LPS-induced cells could be weakened by 3-MA or pcDNA3.1-TLR9 treatment. Injection of AMSC-Exo-342 enhanced autophagy, mitigated kidney injury, suppressed inflammation, and reduced BUN and SCr levels in sepsis-related AKI mouse models.

**Conclusion:**

miR-342-5p transferred by exosomes from miR-342-5p-modified AMSCs ameliorated AKI by inhibiting TLR9 to accelerate autophagy.

**Graphical Abstract:**

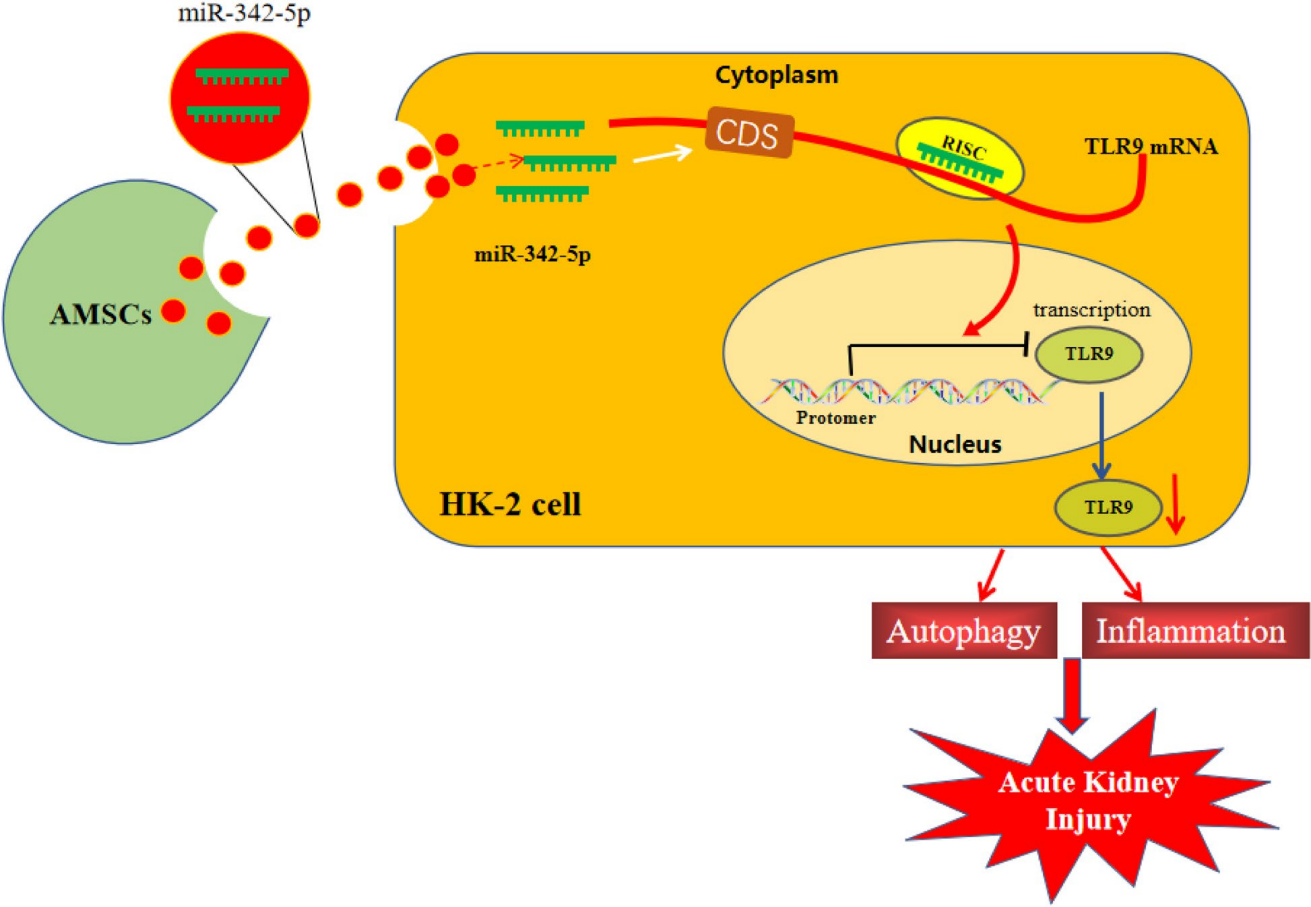

## Introduction

Sepsis is a life-threatening clinical syndrome characterized by organ dysfunction due to a dysregulated host response to infection [1]. Importantly, sepsis is the most essential contributor to the development of acute kidney injury (AKI) in critically ill patients, accounting for approximately 20% ~ 50% of the cases [2, 3]. Sepsis-related AKI is responsible for the increase in chronic comorbidity risk and is associated with extremely high mortality and considerable consumption of healthcare resources [4, 5]. Although the pathophysiology of AKI in sepsis is complex, it is partially affected by nephrotoxins, systemic inflammation, and hemodynamic alterations [6]. Inflammatory cytokines released by injured renal tubular cells are considered to be dominant factors affecting the severity of renal inflammation [7]. Therefore, suppression of the inflammatory reaction may be key for AKI treatment.

Current management therapies for AKI are still limited to supportive care and preventive measures, such as renal transplantation and dialysis [8]. Therefore, it is necessary to improve kidney regeneration following AKI by advancing innovative interventions. As a class of tissue related stem cells, mesenchymal stem cells (MSCs) possess the abilities of multipotential differentiation and self-renewal [9]. Interestingly, the potential therapeutic effects of MSCs on AKI have been verified in previous studies. For instance, human umbilical cord-derived MSCs are highly efficient in the repair of cisplatin-induced AKI [10]. Ischemia/reperfusion injury-induced AKI can be attenuated by bone marrow-derived MSCs through regulation of TSG-6 [11]. Bone marrow-derived MSCs were shown to have a significant role in improving renal function [12, 13], nevertheless, evidence in a recent study reported the beneficial effect of adipose-derived MSCs (AMSCs) exosomes on improving kidney function and structure compared with bone marrow-derived MSCs exosomes [14].

Furthermore, stem cell-derived exosomes are regarded as promising therapeutic options for AKI [15]. Exosomes, small extracellular vesicles of approximately 30 ~ 100 nm, are constitutively released by many cell types and tissues and can be derived from many body fluids such as blood, saliva, and semen [16–18]. Exosomes encompass a variety of bioactive molecules, including nucleic acids, microRNAs (miRNAs), proteins, and lipids, which are transferred to recipient cells through direct membrane fusion, ligand-receptor interaction, or endocytosis [19, 20]. The transfer of miRNAs mediated by exosomes has been studied continuously in recipient cells. For example, exosomes derived from miR-146a-modified AMSCs repress EGR1 to attenuate acute myocardial infarction-induced myocardial damage [21]. A recent study introduced the participation of miR-342-5p in kidney-related diseases and showed that during the renal fibrosis process, miR-342-5p and FoxO3 regulate Ptch1, thus, mediating autophagy [22]. In addition, the regulatory effect of miR-342-5p in inflammation has been demonstrated in previous studies [23, 24]. However, it remains unknown whether miR-342-5p can be carried by exosomes derived from AMSCs to exert a favorable effect in ameliorating AKI.

Toll-like receptors (TLRs) are pattern recognition receptors that are involved in the modulation of adaptive and innate immunity, protecting against microbial invasion [25]. TLR9 recognizes endogenous mitochondrial DNA products released by damaged cells to induce gene transcription, which results in inflammation and apoptosis [26]. Accumulating evidence has revealed that TLR9 contributes to the development of sepsis-related AKI [27, 28]. In the present study, TLR9 was identified as a target gene of miR-342-5p, and miR-342-5p negatively mediated the expression of TLR9. To this end, we investigated whether miR-342-5p carried by AMSC-derived exosomes could interact with TLR9 to protect AKI mice against inflammation and kidney injury.

Here, we studied the performance of exosomal transfer of miR-342-5p from AMSCs in inflammation in both in vitro and in vivo AKI models and identified the relationship among autophagy, inflammation, and the miR-342-5p/TLR9 axis. We propose that miR-342-5p loaded by AMSC-derived exosomes may alleviate the inflammation of HK-2 cells by targeting TLR9 to promote autophagy. Our findings may provide a new approach and therapeutic target for treating sepsis-associated AKI.

## Materials and methods

### Clinical samples

This study was conducted in accordance with the Declaration of Helsinki. The study protocol was approved by the Medical Ethics Committee of Xiangya Hospital Central South University (ethical approval number: 202,106,247). A total of 30 patients (19 male and 11 female) with sepsis-associated AKI, aged 24 ~ 57 years (average age of 39.3 ± 7.8 years), were recruited consecutively from our hospital. The control group in our hospital consisted of 30 healthy volunteers (16 men and 14 women) aged 23 ~ 56 years (average age of 37.4 ± 8.2 years). There was no significant difference between patients with AKI and healthy volunteers with regard to sex and age. AKI cases were identified according to the Kidney Disease Improving Global Outcomes criteria and defined as any of the following symptoms: urine volume ≤ 0.5 mL/kg/h for 6 h; increase in serum creatinine (SCr) to ≥ 50% baseline within 7 days; or increase in SCr by more than 26.5 µmol/L within 48 h. Sepsis is the systemic response to infection, manifested by two or more of the following conditions as a result of infection: (1) body temperature < 36 °C or > 38.5 °C; (2) respiratory rate > 20 breaths/min or PaCO_2_ < 32 mmHg; (3) heart rate > 90 beats/min; and (4) white blood cell count < 4,000/mm^3^ or > 12,000/mm^3^, or > 10% immature band forms. The exclusion criteria were as follows: (1) patients with end-stage renal disease; (2) patients with a history of kidney transplantation; (3) patients who were diagnosed with cancer; (4) patients with acquired immunodeficiency syndrome; and (5) patients who received high-dose steroid therapy. All patients provided written informed consent, and venous blood tests were performed on all patients at the time of diagnosis.

### Isolation and identification of AMSCs [29]

Human AMSCs from Shanghai Zhongqiaoxinzhou Biotech (Shanghai, China) were soaked in MesenCult™-ACF medium (STEMCELL Technologies, CA) supplemented with 2 mM L-glutamine (Thermo Fisher Scientific, Wilmington, DE, USA) and 1% antibiotic antibacterial agent (Thermo Fisher Scientific). The phenotypes of the 3rd to 6th generation of AMSCs were evaluated by flow cytometry analysis (BD Accuri®C6 flow cytometry) after incubation with antibodies against CD29, CD31, CD44, CD45, CD73, CD90, CD105, and HLA-DR (Biolegend, CA, USA). IgG1 served as a negative control. Adipogenic differentiation medium (Invitrogen, Carlsbad, CA, USA), osteogenic differentiation medium (Invitrogen), and chondrogenic differentiation medium (Invitrogen) were used to induce the differentiation of AMSCs. After 14 or 21 days of induction, the cells were stained with Oil Red O dye (Sigma-Aldrich, Merck KGaA, Darmstadt, Germany), Alizarin Red S (Sigma-Aldrich), and Alcian Blue (Sigma-Aldrich).

### Extraction of exosomes by differential centrifugation [30]

After 24 h of cell transfection, the culture medium was replaced with exosome-free fetal bovine serum (FBS)-supplemented medium for 48 h of incubation at 37 °C with 5% CO_2_. Exosomes were harvested by differential centrifugation: the supernatant was obtained, followed by low-speed centrifugation (300 × g for 10 min, 2,000 × g for 10 min) and ultracentrifugation (10,000 × g for 30 min, 100,000 × g for 70 min) at 4 °C. Cell precipitates were resuspended in PBS and then subjected to ultracentrifugation (100,000 × g for 70 min). Then, cell precipitates were resuspended in 200 µL PBS prior to the detection of protein concentration using the bicinchoninic acid (BCA) method or storage at -80 °C for later use. Quantitative analysis of exosomes was performed using the BCA protein assay kit (Beyotime Biotech, Shanghai, China) in accordance with the manufacturer’s instructions.

### Observation of the morphology of exosomes by transmission electron microscope (TEM) [31]

Exosome suspensions (10 µL) were diluted with PBS and added to a 2 mm formvar-coated copper grid for 1 min of incubation at room temperature. After removing excess liquid with filter paper, exosomes were negatively stained with 3% (w/v) sodium phosphotungstate solution (Sigma-Aldrich) for 5 min. Then, exosomes were washed with PBS, dried naturally in the air prior to observation, and photographed using a TEM (H-8100, Hitachi, Tokyo, Japan) at 80 ~ 120 kV (Scare bar = 0.2 μm).

### Nanosight tracking analysis [32]

Exosomes were diluted 1:100, and 1 mL exosome solution was added to a quartz colorimetric utensil. The particle size was determined using a dynamic light-scattering instrument (Nano S90, Marvin, UK).

### Fluorescence labeling of exosomes [33]

AMSCs were labeled with 1.25 µM lipophilic carbocyanine DiLC_16_ (3) for 10 min at 37 °C, followed by washing and resuspension in fresh culture medium for 48 h. Exosomes labeled by fluorescence were added into HK-2 cells for incubation of 6 h and the cells were fixed in formaldehyde and sealed. Images were captured using a fluorescence microscope.

### Cell culture and cell transfection [34]

The human renal proximal tubular epithelial cell line HK-2 (National Collection of Authenticated Cell Cultures, Shanghai, China) was immersed in Dulbecco’s modified Eagle medium (Gibco, Grand Island, NY, USA) containing 10% FBS, 1% penicillin, and 1% streptomycin for cell culture in an incubator at 37 °C and 5% CO_2_. For the construction of sepsis cellular models, HK-2 cells were induced by 1 µg/mL lipopolysaccharide (LPS, Sigma-Aldrich) for 24 h. To investigate the effect of autophagy on sepsis, HK-2 cells were treated with autophagy activator rapamycin (Rapa, 10 nM, Sigma-Aldrich) or autophagy inhibitor 3-Methyladenine (3-MA, 10 µM, Sigma-Aldrich) for 30 min before LPS treatment. For incubation with exosomes (20 µg), HK-2 cells were treated with LPS (1 µg/mL) for 24 h before being treated by AMSC-Exo for 24 h.

The LPS-induced cells were placed into plates and incubated for 16 h at 37 °C with 5% CO_2_ before cell transfection. Next, cells were transfected with miR-342-5p mimic, miR-342-5p inhibitor, miR-67 mimic, miR-67 inhibitor (50 nM), pcDNA3.1-TLR9, si-TLR9 (2 µg), or their negative controls (GenePharma, Shanghai, China) using the LipoFiterTM transfection reagent (Hanbio Biotech, Shanghai, China) following the manufacturer’s instructions. Three replicates were established, and the following experiments were carried out 24 h after transfection.

### Lentiviral Transfection

The AMSCs (1 × 10^6^ cells) were immersed in 10 mL MesenCultTM-ACF medium overnight. Then, AMSCs were transfected with LV-miR-342-5p (lentiviral vector overexpressing miR-342-5p) and LV-miR-67 (lentiviral vector overexpressing cel-miR-67) (MOI = 10.0). Cel-miR-67 was chosen as the control because it was demonstrated to have no binding sites with mRNAs in humans. Twenty-four hours after transfection, AMSCs were collected for qRT-PCR analysis.

### Animals [34]

All experiments involving animals were performed according to the protocol approved by the Animal Experiment Ethics Committee of Central South University (ethical approval number: 202,106,247). Six-to-eight-week-old male C57BL/6J mice (*n* = 36) were purchased from the Shanghai Experimental Animal Center of the Chinese Academy of Sciences. All mice were bred and maintained under specific pathogen-free conditions at the humidity of 40 ~ 60% for 1 week with a 12 h light-dark cycle. Water and standard laboratory food were provided ad libitum.

The sepsis-associated AKI mouse models (the CLP group, *n* = 6) were established by cecal ligation and puncture (CLP). Briefly, mice were anesthetized with ketamine (100 mg, Ketavet®)/xylazine (200 mg/kg, Rompun®) after being weighed. A 1 ~ 1.5 cm incision was made in the middle of the lower abdomen, and the cecum was carefully separated. The distal 1/3 of the cecum was ligated with a 4 − 0 silk ligature. The end of the cecum was punctured twice with a 25-gauge sterile needle, and a small amount of feces was gently squeezed out to ensure that the punctures were unblocked. The cecum was then put back into the abdomen, and the incision was sutured. Mice in the sham group (*n* = 6) underwent surgery without ligation and puncture. Mice in the CLP + AMSC-Exo-67 group (*n* = 6) or the CLP + AMSC-Exo-342 group (*n* = 6) were injected with 100 µg AMSC-Exo-67 or AMSC-Exo-342 by tail vein 4 h after CLP. In the CLP + Rapa group (*n* = 6), 4 mg/kg Rapa (Sigma-Aldrich) was intraperitoneally injected into mice 6 h before surgery, and 200 µL normal saline was injected into mice via the tail vein 4 h after surgery. Mice in the CLP + 3-MA + AMSC-Exo-342 group were administered 20 mg/kg 3-MA by intraperitoneal injection 6 h before operation and injected with 100 µg AMSC-Exo-342 by tail vein 4 h after CLP. Kidney tissues and blood samples from each group were obtained 72 h later.

### Quantitative Reverse transcription-polymerase Chain Reaction (qRT-PCR)

Total RNA from cells or tissues was extracted according to the instructions included with TRIzol reagent (Invitrogen), and the concentration and quality of total RNA were determined. After adjustment to an appropriate concentration, total RNA was reverse transcribed using a reverse transcription kit (TaKaRa, Tokyo, Japan). Random primers were applied as reverse transcription primers, and the operation was performed according to the instructions included with the reverse transcription kit and random primers. Gene expression was assessed using a LightCycler 480 fluorescence quantitative PCR instrument (Roche, Indianapolis, IN, USA). The reaction conditions were consistent with the instructions for the fluorescent quantitative PCR kit (SYBR Green Mix, Roche Diagnostics, Indianapolis, IN, USA). The reaction conditions were initial denaturation at 95 °C for 5 min, followed by 40 cycles of denaturation at 95 °C for 10 s, annealing at 60 °C for 10 s, and extension at 72 °C for 20 s. Three independent experiments were run in parallel. The quantification of mRNA was normalized to that of GAPDH, whereas that of miRNAs was normalized to U6. All primers used are listed in Table 1.

**Table 1:** Primer sequence information

Name of primer	Sequences
*U6*-F	CTCGCTTCGGCAGCACA
*U6*-R	ACGCTTCACGAATTTGCGT
Hsa-miR-342-5p-F	ACTAGGGGTGCTATCTGTGA
Hsa-miR-342-5p-R	GTGCAGGGTCCGAGGT
*GAPDH*-F	GCAAGGATGCTGGCGTAATG
*GAPDH*-R	TACGCGTAGGGGTTTGACAC
TLR9-F	CACCCACTCCACTTCATGGG
TLR9-R	GGAGCAGGAAGGGAAAGTCG

*F *forward primer, *R *reverse primer

### Western blot [35]

Cells or tissues were lysed with RIPA lysis buffer (Beyotime) and centrifuged to obtain protein samples. GAPDH served as an internal reference. The protein concentration was determined using a BCA kit (Beyotime), and the corresponding volume of protein was mixed with loading buffer (Beyotime), followed by 3 min of denaturation in a boiling water bath. SDS-PAGE (10%) was performed in compliance with the directions for the SDS-PAGE preparation kit (Beyotime) and was then applied to separate proteins. Electrophoresis was conducted at 80 V, and then for 1 ~ 2 h at 120 V once bromophenol blue reached the separation gel. Then, the proteins were transferred onto membranes at 300 mA for 60 min in an ice bath. The membranes were rinsed for 1 ~ 2 min with washing solution, sealed in blocking solution at room temperature for 60 min, or inactivated overnight at 4℃. The membranes were incubated with primary antibodies against rabbit anti-human GAPDH (1:1000, Cell Signaling Technology [CST], Boston, USA), CD9 (1:500, Santa Cruz, Texas, USA), CD81 (1:500; Santa Cruz), CD63 (1:1000, Abcam, MA, USA), LC-3 (1:2000, Abcam), p62 (1:10000, Abcam), Beclin-1 (1:1000, CST), and TLR9 (1:1000, Abcam) at room temperature on a shaking table for 1 h. Following primary incubation, the membranes were washed with the washing solution for 3 × 10 min and then incubated with horseradish peroxidase-conjugated goat anti-rabbit IgG (1:5000, Beijing ComWin Biotech Co., Ltd., Beijing, China) for 1 h at room temperature. The membranes were washed for 3 × 10 min with washing solution and subjected to color development. A chemiluminescence imaging system (Bio-Rad, Hercules, CA, USA) was used for these observations.

### Dual-luciferase reporter assay [36, 37]

TargetScan (http://www.targetscan.org/mamm_31/) was used to predict the binding sites of miR-342-5p and TLR9. The mutated and wild-type sequences (mut-TLR9 and wt-TLR9) were designed and synthesized in accordance with the predicted results, and cloned into a luciferase reporter vector (pGL3-Basic, Promega, Madison, WI, USA). The vectors were co-transfected with miR-342-5p mimic or mimic-NC (30 nM, GenePharma) into HEK293T cells. After transfection, the dual-luciferase reporter assay kit (Promega) was used to measure the fluorescence intensity of cells in each group and to determine the binding of miR-342-5p to TLR9. Cells were grouped into the mimic + mut-TLR9 group, mimic + wt-TLR9 group, mimic NC + mut-TLR9 group, and mimic NC + wt-TLR9 group. Three replicates were used for this test.

### Hematoxylin-eosin (H&E) staining [38]

Kidney tissues were fixed in 10% paraformaldehyde for 24 h and then subjected to gradient dehydration and paraffin embedding. H&E staining was performed after 4 μm thick paraffin sections were prepared.

### Immunofluorescence [39]

Cells were fixed in 4% paraformaldehyde for 15 min, permeabilized with 1% Triton X-100 for 25 min at room temperature, and washed three times with PBS. After 45 min of inactivation in 5% bovine serum albumin, the cells were incubated with the primary antibody overnight at 4 °C, followed by 3 × 5 min of PBS wash. The cells were then exposed to a secondary antibody (DyLight® 650, goat anti-rabbit IgG) at room temperature for 2 h. The nuclei of cells were stained with DAPI (Beyotime) and visualized under a microscope (Nikon, Japan).

### TEM [38]

HK-2 cells were starved with Earle’s Balanced Salt Solution for 30 min, washed with PBS, and centrifuged for 10 min. The cells were then fixed in glutaraldehyde for 2 ~ 4 h and washed with 0.1 mol/L PBS for 1 h at 4 °C. The washing liquid was changed 3 times during this process. Cells were immersed in osmium tetroxide fixative for 1 ~ 2 h at 0 ~ 4℃, followed by exposure to acetone (30%, 70%, and 90%, each for 10 min) and pure acetone (3 × 10 min). After being embedded in the embedding medium, the cells were polymerized in an oven at 37 °C for 12 h and 60 °C for 24 h. For examination via TEM (H-8100, Hitachi, Tokyo, Japan), sections of 60 nm were produced using a Reichert-Jung Ultracut E ultramicrotome (Leica Microsystems).

### Enzyme-linked Immunosorbent Assay (ELISA)

The serum of humans and mice and the supernatant of HK-2 cells were collected. ELISA kits were used to detect the levels of blood urea nitrogen (BUN) (YS02947B, Jiya biological and technology co., Ltd, Shanghai, China), SCr (FT-P9S3176X, Fantai biological and technology co., Ltd, Shanghai, China), tumor necrosis factor (TNF)-α (ab285312, ab285312, Abcam), interleukin (IL)-1β (ab214025, ab255730, Abcam), IL-6 (ab178013, ab222503, Abcam), and monocyte chemoattractant protein (MCP)-1 (ab179886, ab208979, Abcam) in the serum and cell supernatant.

### Statistical analysis

Three sets of samples were used for each group in each experiment. Statistical analysis was conducted using GraphPad software (version 7.0). Data were expressed as mean ± standard deviation (SD). A *t*-test was employed for comparisons between two groups. Comparisons among multiple groups were analyzed by one-way analysis of variance (ANOVA) and confirmed by Tukey’s multiple comparison test. *P* values were considered significant at *P* < 0.05.

## Results

### High expression of TLR9 and low expression of miR-342-5p in patients with AKI and AKI cellular models

**Fig. 1:**
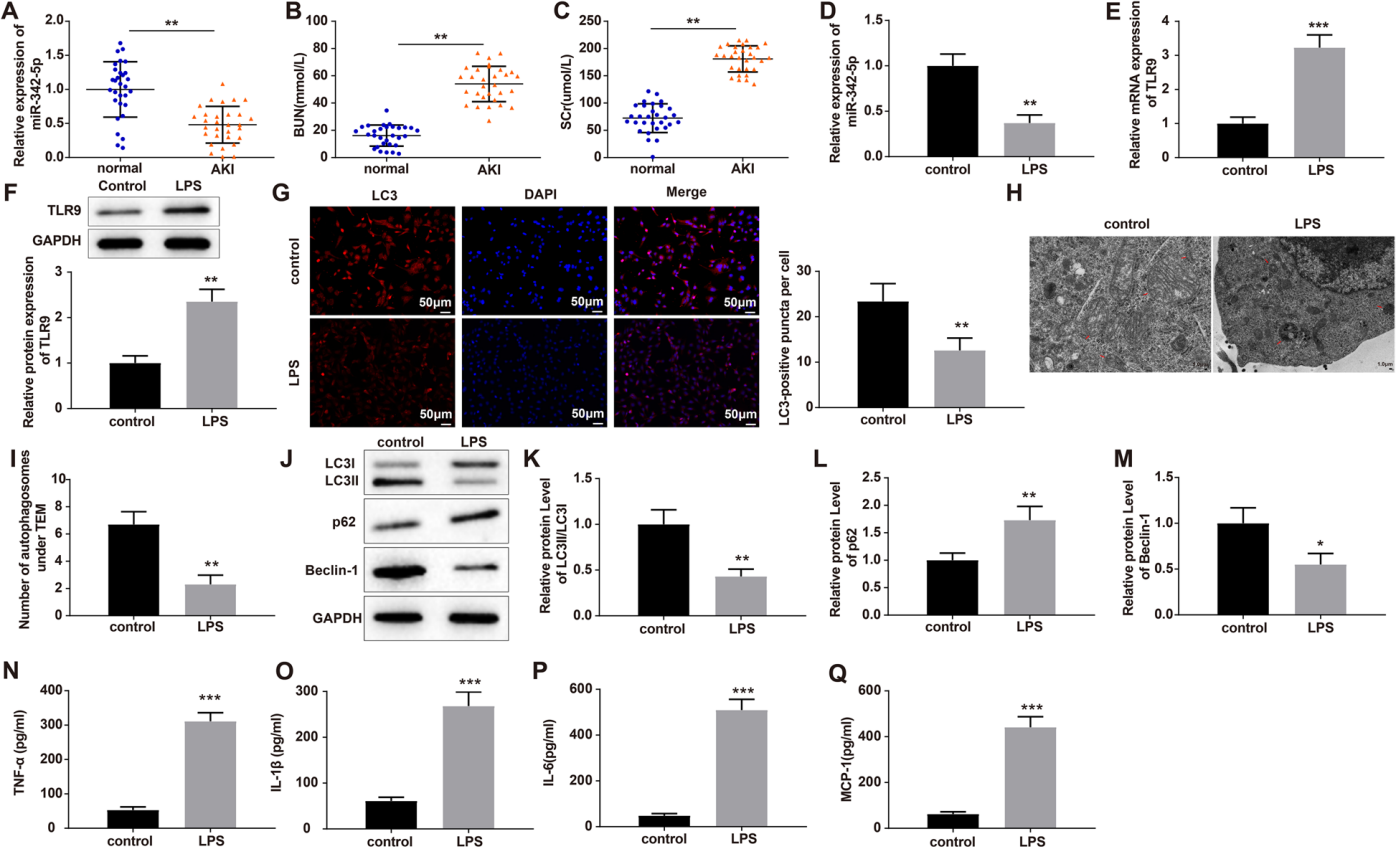
Expressions of miR-342-5p and TLR9 in AKI. Notes: qRT-PCR measured the mRNA expression of miR-342-5p in the serum of healthy volunteers and sepsis-associated AKI patients (**A**). The levels of BUN (**B**) and SCr (**C**) in serum of healthy volunteers and AKI patients were detected by ELISA. After construction of AKI cell models, the expressions of miR-342-5p (**D**) and TLR9 (**E**, **F**) were examined by qRT-PCR and western blot. Immunofluorescence assessed the fluorescence intensity of LC3 in HK-2 cells (**G**), and TEM observed the number of autophagosomes in HK-2 cells (**H**, **I**). Then, western blot was used to inspect the expressions of autophagy-related proteins LC3-II/LC3-I, p62 and Beclin-1 (**J**-**M**), and ELISA to evaluate inflammation-related factors TNF-α, IL-1β, IL-6, and MCP-1 in the supernatant of HK-2 cells (N-Q); *N* = 3, ^**^ *P* < 0.01, compared to the Normal/control group; ^*^ *P* < 0.05, ^**^ *P* < 0.01, ^***^ *P* < 0.001, compared to the Control group; AKI, acute kidney injury. Data were analyzed by the two-tailed *T* test. Data of in vitro experiments were generated from three independent experiments

To probe the function of miR-342-5p in sepsis-induced AKI, we collected serum of 30 patients with sepsis-associated AKI and 30 healthy volunteers and detected miR-342-5p expression by qRT-PCR. Results showed that miR-342-5p expression in the serum of patients with sepsis-related AKI was remarkably lower than that in healthy volunteers (Fig. 1A, *P* < 0.01). To evaluate the difference in renal function between patients with sepsis-associated AKI and healthy volunteers, ELISA was used to analyze the content of BUN and SCr, which revealed that patients with sepsis-associated AKI possessed higher levels of BUN and SCr in comparison to the healthy volunteers (Fig. 1B, C, *P* < 0.01).

Next, a cellular model of sepsis in HK-2 cells was established by LPS induction. Displayed by qRT-PCR, LPS treatment suppressed miR-342-5p expression in HK-2 cells (Fig. 1D, *P* < 0.01). Further qRT-PCR and western blot showed that LPS treatment increased TLR9 expression in HK-2 cells (Fig. 1E-F, *P* < 0.01). The fluorescence intensity of autophagy marker light chain (LC)3 in the LPS group was lower than that in the control group, as shown by immunofluorescence staining (Fig. 1G, *P* < 0.01). The number of autophagosomes in HK-2 cells was observed under TEM, and cells in the LPS group had fewer autophagosomes than cells in the control group (Fig. 1H, I, P < 0.01). Additionally, western blot analysis showed that LPS induction led to elevated p62 (*P* < 0.01), repressed the ratio of LC3-II/LC3-I (*P* < 0.01), and diminished Beclin-1 levels (*P* < 0.05) in HK-2 cells (Fig. 1J-M).

The findings of the ELISA illustrated that the LPS group showed higher levels of TNF-α, IL-1β, IL-6, and MCP-1 in the supernatant of HK-2 cells than in the control group (Fig. 1N-Q, *P* < 0.001). These data indicated that there were downregulated miR-342-5p, upregulated TLR9, and suppressed autophagy in AKI.

### Identification of AMSCs

**Fig. 2:**
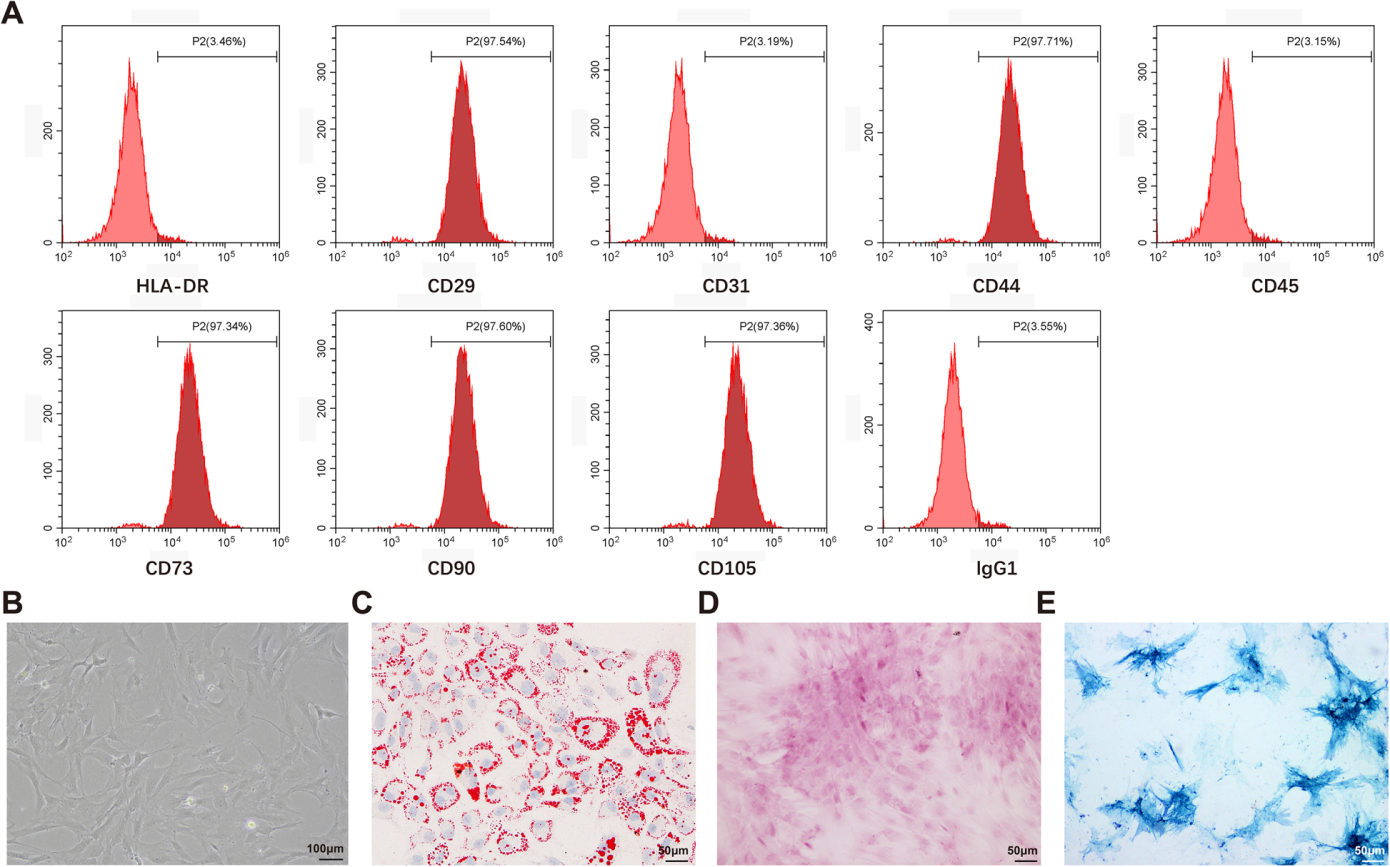
Induction of extracted cells for differentiation into adipocytes, osteocytes, and chondrocytes to identify their differential potential as stem cells. Notes: The surface markers of AMSCs (CD29, CD44, CD73, CD90, CD105, CD31, CD45, and HLA-DR) were analyzed by flow cytometry (**A**). The morphology of AMSCs was observed (**B**). Following adipogenic, osteogenic, or chondrogenic induction (14 days/21 days/21 days), AMSCs underwent Oil red O, Alizarin Red S, and Alcian Blue staining (**C**-**E**); Scale bar = 50 μm; AMSCs, adipose-derived mesenchymal stem cells

To determine whether AMSC-secreted exosomes can be used as an effective carrier for miR-342-5p to alleviate AKI, the AMSCs were cultured, and the surface antigens of the AMSCs were analyzed by flow cytometry. The findings revealed that the isolated cells showed positive expression of CD29, CD44, CD73, CD90, and CD105, and negative expression of CD31, CD45, and HLA-DR (Fig. 2A). After 24 h of cell culture, a small number of cells adhered to the wall, showing the appearance of fibroblasts with filaments at both ends (Fig. 2B). Then, the AMSCs were allowed to differentiate. After adipogenic differentiation of AMSCs for 14 days in vitro, fat droplets were labeled as red by Oil Red O staining (Fig. 2C). AMSCs were stained red with Alizarin Red S after 21 days of osteogenic differentiation (Fig. 2D). After 21 days of chondrogenic induction, Alcian blue-positive cells were observed in the AMSCs (Fig. 2E). The above results suggested that the isolated AMSCs possessed typical characteristics of stem cells with multidirectional differentiation potential and could be used in subsequent experiments.

### AMSC-derived Exosomes Deliver miR-342-5p to HK-2 Cells

**Fig. 3:**
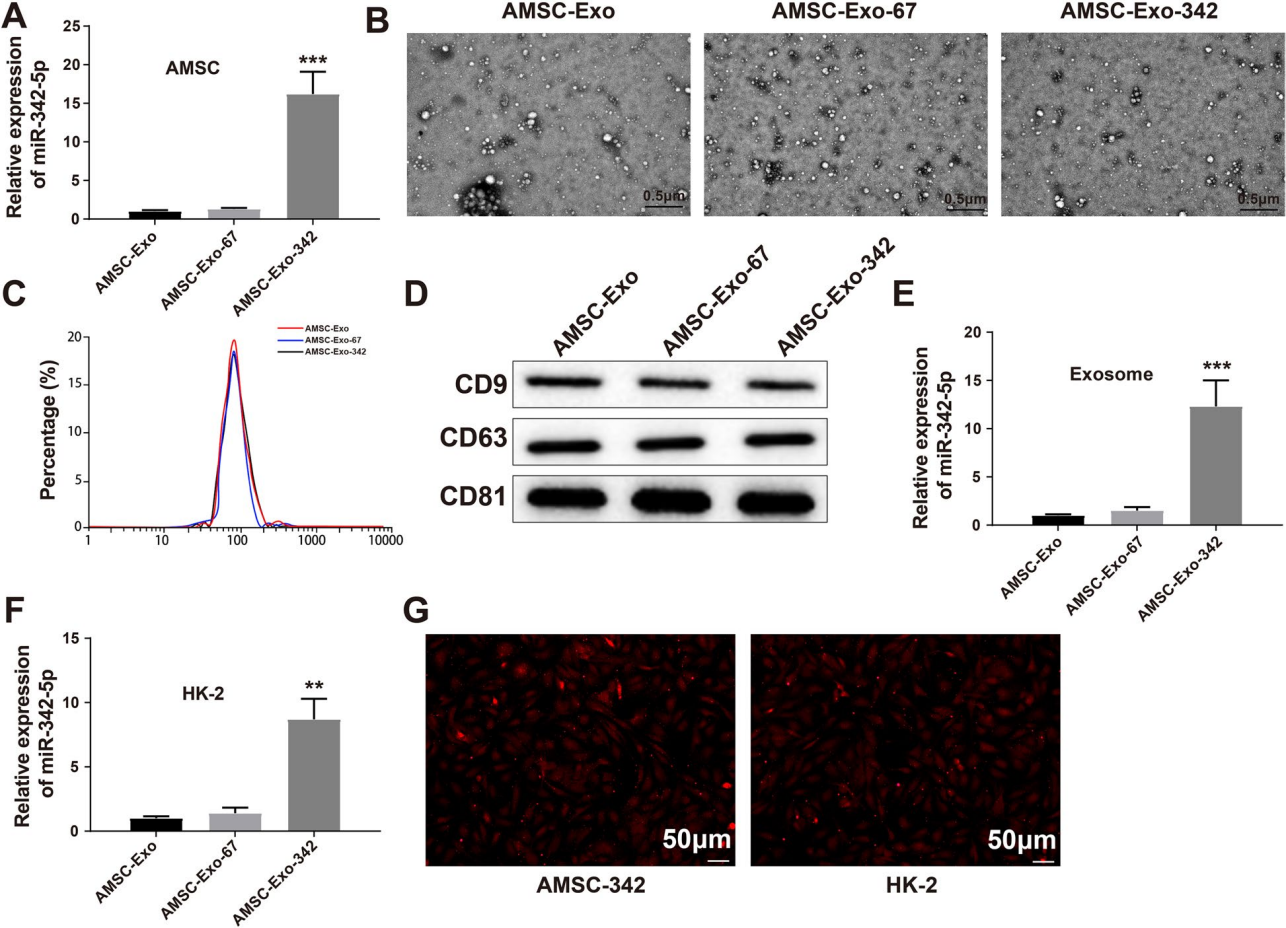
Transfer of AMSC-Exo-mediated miR-342-5p into HK-2 cells. Notes: After AMSCs were transfected with LV-miR-342-5p or LV-miR-67, qRT-PCR was used to detect the expression of miR-342-5p in AMSCs (**A**). The morphology of exosomes was observed by TEM (**B**), and the particle size of exosomes was measured by Nanosight tracking analysis (**C**). Western blot was utilized to examine the protein expressions of exosome marker proteins CD9, CD63, and CD81 (**D**). The mRNA expression of miR-342-5p in AMSC-derived exosomes was measured by qRT-PCR (**E**). Then, HK-2 cells were subjected to 50 ng/µL of AMSC-Exo, AMSC-Exo-342, or AMSC-Exo-67 treatment for 24 h. qRT-PCR was used to detect the expression of miR-342-5p in HK-2 cells (**F**). ^**^ *P* < 0.01, ^***^ *P* < 0.001, compared to the AMSC-Exo-67 group, *N* = 3. The red fluorescence in HK-2 cells incubated with DiLC_16_ (3)-labeled AMSC-Exo-342 was observed under a fluorescence microscope (**G**); AMSCs, adipose-derived mesenchymal stem cells; Exo, exosome; TEM, transmission electron microscope

Subsequently, lentiviral vectors overexpressing miR-342-5p (LV-miR-342-5p) or lentiviral vectors overexpressing cel-miR-67 (LV-miR-67; as a control) were transfected into AMSCs to obtain miR-342-5p-modified AMSCs (AMSC-342) and miR-67-modified AMSCs (AMSC-67). qRT-PCR results showed that transfection with LV-miR-342-5p enhanced the level of miR-342-5p in AMSCs (Fig. 3A, *P* < 0.001). Twenty-four hours after transfection, exosomes were extracted from the supernatant of the AMSCs. TEM revealed small round or oval vesicles with uniform size and shape (Fig. 3B). Nanosight tracking analysis showed that the particle diameter of exosomes ranged from 50 to 150 nm, with a peak at 80 nm (Fig. 3C). Furthermore, western blotting revealed high expression of CD9, CD63, and CD81 (Fig. 3D). These findings implied that the exosomes were successfully extracted.

The levels of miR-342-5p in AMSC-derived exosomes were measured by qRT-PCR. Higher miR-342-5p expression was observed in the AMSC-Exo-342 group than in the AMSC-Exo-67 group (Fig. 3E, *P* < 0.001). To observe whether AMSC-derived exosomes can be taken up by HK-2 cells, the HK-2 cells were exposed to 50 ng/µL of AMSC-Exo, AMSC-Exo-342, or AMSC-Exo-67 for 24 h. Elevated miR-342-5p levels were observed in cells from the AMSC-Exo-342 group as compared to those in the AMSC-Exo-67 group, as shown by qRT-PCR (Fig. 3F, *P* < 0.01). Then, to observe whether AMSC-derived exosomes can be taken up by HK-2 cells, the HK-2 cells were cultured with DiLC_16_ (3)-labeled AMSC-Exo-342. The results demonstrated that red fluorescence was detected in HK-2 cells after incubation with DiLC_16_ (3)-labeled AMSC-Exo-342 (Fig. 3G). Collectively, these results indicated that AMSC-derived exosomes could be taken up by HK-2 cells and that AMSC-derived miR-342-5p could be successfully transferred into HK-2 cells via exosomes.

### AMSC-Exo-342 Mitigates LPS-induced Inflammation in HK-2 Cells by Promoting cell Autophagy

**Fig. 4:**
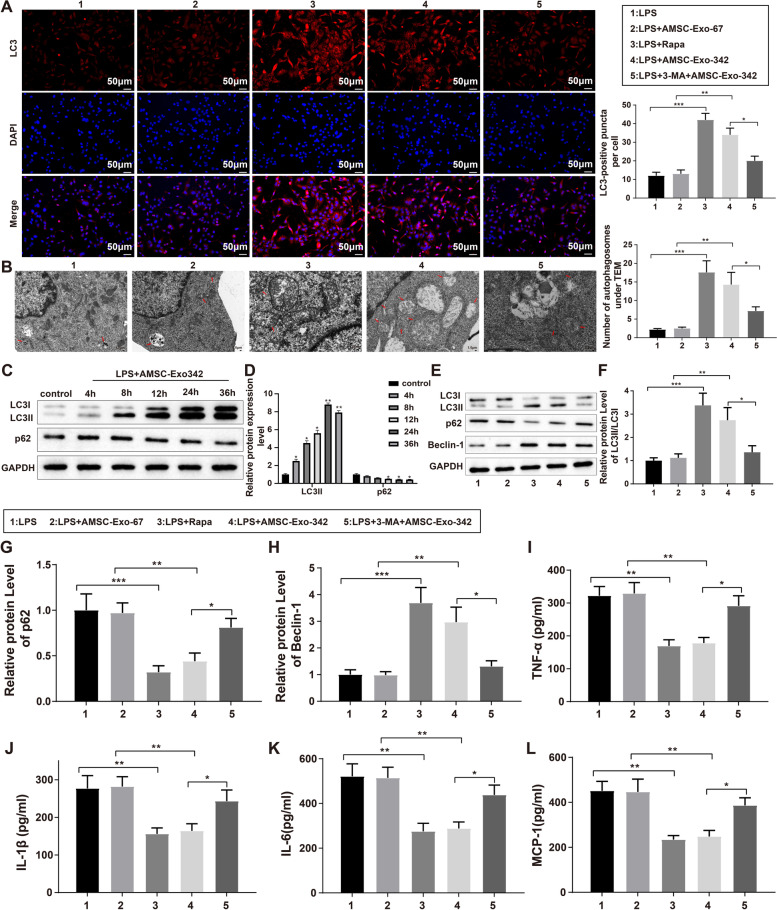
AMSC-Exo-342 promotes cell autophagy to ameliorate LPS-induced cell inflammation. Notes: The HK-2 cells received autophagy activator Rapa or autophagy inhibitor 3-MA prior to LPS induction and exposure to AMSC-Exo-67 or AMSC-Exo-342. The fluorescence intensity of LC3 was measured by immunofluorescence staining (**A**), and the number of autophagosomes in HK-2 cells was observed by TEM (**B**). The expressions of LC-3II/LC-3I and p26 in cells in the LPS + AMSC-Exo-342 group at 4 h, 8 h, 12 h, 24 h, and 36 h were measured by western blot (**C**, **D**). The protein expressions of autophagy-related proteins LC3-II/LC3-I, p62, and Beclin-1 were detected by western blot (**F**-**H**). ELISA analyzed the contents of inflammatory cytokines TNF-α (I), IL-1β (J), IL-6 (K), and MCP (L) in the supernatant of HK-2 cells. ^*^ *P* < 0.05, ^**^ *P* < 0.01, ^***^ *P* < 0.001, compared to the Control, LPS, LPS + AMSC-Exo-67, or LPS + AMSC-Exo-342 group; AMSCs, adipose-derived mesenchymal stem cells; Exo, exosome; LPS, lipopolysaccharide; Rapa, Rapamycin; 3-MA, 3-Methyladenine; TEM, transmission electron microscope. Data were analyzed using one-way analysis of variance and Tukey test was used for post hoc analysis. Data were generated from three independent experiments

To determine whether AMSC-Exo-342 could be implicated in sepsis-associated AKI by acting on cell autophagy, HK-2 cells were exposed to 10 nM autophagy activator Rapa or autophagy inhibitor 3-MA before LPS induction and treatment with AMSC-Exo-67 or AMSC-Exo-342. Immunofluorescence results showed that pretreatment with Rapa or post-treatment with AMSC-Exo-342 enhanced the fluorescence intensity of LC3 in LPS-treated HK-2 cells (Fig. 4A, LPS group vs. LPS + Rapa group, *P* < 0.001; LPS + AMSC-Exo-67 group vs. LPS + AMSC-Exo-342 group, *P* < 0.01). However, the LPS + 3-MA + AMSC-Exo-342 group showed lower fluorescence intensity of LC3 than the LPS + AMSC-Exo-342 group (Fig. 4A, *P* < 0.05). Additionally, the number of autophagosomes in HK-2 cells observed by TEM exhibited the same trend as the fluorescence intensity of LC3 (Fig. 4B). After LPS-treated cells were incubated with AMSC-Exo-342, western blotting was used to measure the ratio of LC3-II/LC-3I and the expression of p62. After incubation for 24 h, the LC3-II/LC-3I ratio reached its maximum, and p62 expression reached the lowest level; therefore, the optimal time for cell incubation to induce cell autophagy was 24 h. In this regard, further experiments were conducted on cells after LPS + AMSC-Exo-342 treatment for 24 h (Fig. 4C, D, *P* < 0.05).

Western blot was used to analyze the expression of autophagy-related proteins, and the findings showed that compared with the LPS group/LPS + AMSC-Exo-67 group, the LPS + Rapa group/LPS + AMSC-Exo-342 possessed elevated levels of LC3-II/LC3-I ratio and Beclin-1 and decreased p62 levels (Fig. 4E-H, *P* < 0.001/*P* < 0.01), while higher p62 expression and lowerLC3-II/LC3-I and Beclin-1 expression were observed in the LPS + 3-MA + AMSC-Exo-342 group rather than in the LPS + AMSC-Exo-342 group (Fig. 4E-H, *P* < 0.05). The ELISA results showed that AMSC-Exo-342 transfection or Rapa treatment in HK-2 cells significantly inhibited LPS-enhanced inflammatory response (Fig. 4I-L, *P* < 0.01), while 3-MA weakened the inhibitory effect of AMSC-Exo-342 on the release of inflammatory cytokines in LPS-induced HK-2 cells (Fig. 4I-L, *P* < 0.05). These findings suggested that AMSC-Exo-342 may alleviate LPS-induced inflammation in HK-2 cells by accelerating cell autophagy.

### miR-342-5p Negatively Mediates TLR9

**Fig. 5:**
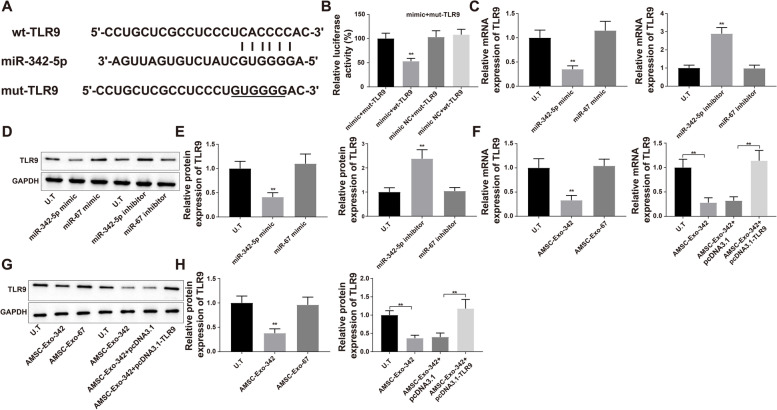
TLR9 can be modulated by miR-342-5p. Notes: The binding site of miR-342-5p and TLR9 was predicted by TargetScan, and the 3’-UTR region of miR-342-5p and TLR9 was constructed (**A**). Dual-luciferase reporter assay confirmed the interaction between TLR9 and miR-342-5p (**B**). Subsequently, the HK-2 cells were transfected with miR-342-5p mimic, miR-67 mimic, miR-342-5p inhibitor, or miR-67 inhibitor. qRT-PCR (**C**) and western blot (**D**, **E**) were used to detect the mRNA and protein expressions of TLR9. After HK-2 cells were exposed to AMSC-Exo-342 or transfected with pcDNA3.1-TLR9 prior to treatment with AMSC-Exo-342, the mRNA and protein expressions of TLR9 in HK-2 cells were assessed by qRT-PCR (**F**) and western blot (**G**, **H**); *N* = 3. ^**^ *P* < 0.01, compared to the mimic NC + wt-TLR9 group, miR-67 mimic group, miR-67 inhibitor group, AMSC-Exo-67 group, or AMSC-Exo-342 + pcDNA3.1 group; UTR, untranslated region; AMSCs, adipose-derived mesenchymal stem cells; Exo, exosome. Data were analyzed using one-way analysis of variance and Tukey test was used for post hoc analysis. Data were generated from three independent experiments

The online software TargetScan predicted that miR-342-5p had binding sites for TLR9 (Fig. 5A). Then, mutated and wild-type plasmids containing the 3UTR region of TLR9 were constructed for the dual-luciferase reporter assay. The luciferase activity of the mimic + wt-TLR9 group was notably lower than that of the mimic + mut-TLR9 and mimic NC + wt-TLR9 groups (Fig. 5B, *P* < 0.01). Subsequently, HK-2 cells were transfected with miR-342-5p mimic, miR-67 mimic, miR-342-5p inhibitor, or miR-67 inhibitor. The results of qRT-PCR and western blotting showed that overexpression of miR-342-5p suppressed the mRNA and protein expression of TLR9, whereas inhibition of miR-342-5p enhanced TLR9 levels (Fig. 5C-E, *P* < 0.01). HK-2 cells were then exposed to AMSC-Exo-342 or transfected with pcDNA3.1-TLR9 plus AMSC-Exo-342. qRT-PCR and western blot analyses demonstrated that incubation with AMSC-Exo-342 decreased TLR9 expression, while transfection with pcDNA3.1-TLR9 reversed AMSC-Exo-342-mediated inhibition of TLR9 expression (Fig. 5F-H, *P* < 0.01). These data indicated that miR-342-5p may target and negatively regulate TLR9 expression.

### AMSC-Exo-342 Promotes Autophagy by Targeting TLR9 to Reduce LPS-induced Inflammation in HK-2 Cells

**Fig. 6:**
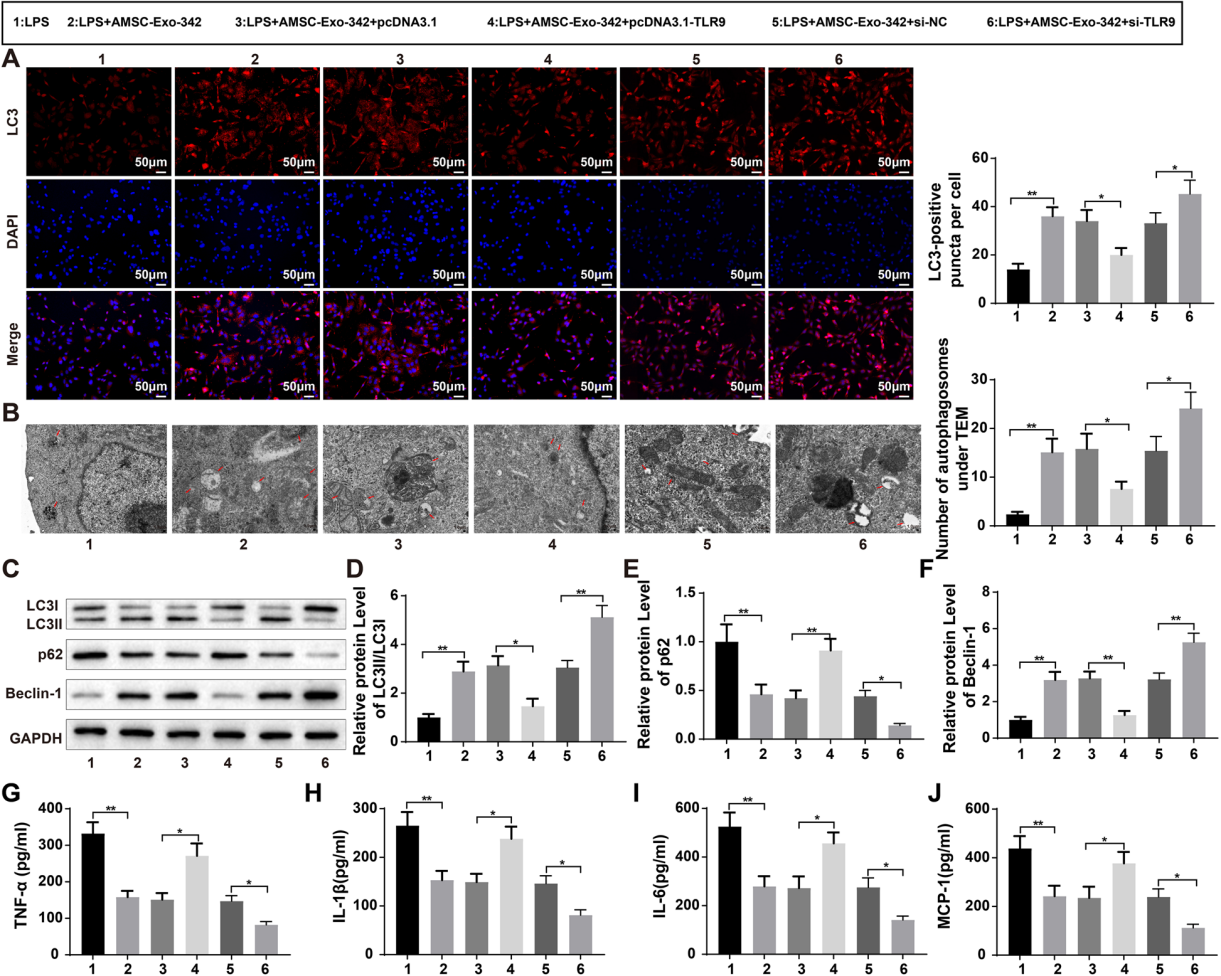
AMSC-Exo-342 ameliorates LPS-induced inflammation by targeting TLR9 to promote autophagy. Notes: After LPS exposure, HK-2 cells were given AMSC-Exo-342 or transfected with pcDNA3.1-TLR9 or si-TLR9 followed by incubation with AMSC-Exo-342. Immunofluorescence staining method was used to detect the fluorescence intensity of LC3 in LPS-induced HK-2 cells (**A**), and TEM to observe the number of autophagosomes (**B**). The protein expression levels of autophagy-related proteins LC3-II/LC3-I (**D**), p62 (**E**), and Beclin-1 (**F**) were measured by western blot (**C**). Then, the levels of inflammatory cytokines TNF-α (**G**), IL-1β (**H**), IL-6 (**I**), and MCP-1 (**J**) in the supernatant of LPS-induced HK-2 cells were inspected by ELISA; *N* = 3. ^*^ *P* < 0.05, compared to the LPS + AMSC-Exo-342 + pcDNA3.1 or LPS + AMSC-Exo-342 + si-NC group; ^**^ *P* < 0.01, compared to the LPS group; AMSCs, adipose-derived mesenchymal stem cells; Exo, exosome; LPS, lipopolysaccharide; TEM, transmission electron microscope. Data were analyzed using one-way analysis of variance and Tukey test was used for post hoc analysis. Data were generated from three independent experiments

Following LPS induction, HK-2 cells were incubated with AMSC-Exo-342 or transfected with pcDNA3.1-TLR9, si-TLR9, or their negative controls prior to exposure to AMSC-Exo-342. Immunofluorescence assay was used to detect the fluorescence intensity of LC3 to evaluate autophagy, and results indicated that exposure to AMSC-Exo-342 potentiated the fluorescence intensity of LC3 (Fig. 6A, *P* < 0.01), and AMSC-Exo-342 + si-TLR9 treatment further increased the fluorescence intensity of LC3 (Fig. 6A, *P* < 0.05); however, overexpression of TLR9 weakened the effects of AMSC-Exo-342 on autophagy in sepsis cellular models (Fig. 6A, *P* < 0.05). Similarly, TEM observation displayed that AMSC-Exo-342 or AMSC-Exo-342 + si-TLR9 treatment increased the number of autophagosomes (Fig. 6B, *P* < 0.01, *P* < 0.05), while overexpression of TLR9 reversed the elevated autophagosomes induced by AMSC-Exo-342 (Fig. 6B, *P* < 0.05). Furthermore, western blot analysis for assessment of the expression of autophagy-related proteins revealed that after AMSC-Exo-342 or AMSC-Exo-342 + si-TLR9 treatment, the ratio of LC3-II/LC3-I and Beclin-1 expression in LPS-induced HK-2 cells was strikingly elevated, and the level of p62 was decreased (Fig. 6C-F, *P* < 0.01), whereas transfection with pcDNA3.1-TLR9 reduced the autophagy enhanced by AMSC-Exo-342 (Fig. 6C-F, P < 0.01). ELISA revealed pronounced increases in the levels of inflammatory cytokines in the LPS + AMSC-Exo-342 + pcDNA3.1-TLR9 group (Fig. 6G-J, *P* < 0.05, vs. LPS + AMSC-Exo-342 + pcDNA3.1 group) and prominent decreases in these cytokines in the LPS + AMSC-Exo-342 or LPS + AMSC-Exo-342 + si-TLR9 group (Fig. 6G-J, *P* < 0.01, vs. LPS or LPS + AMSC-Exo-342 + si-NC group). These findings suggested that AMSC-Exo-342 mitigated LPS-induced inflammation in HK-2 cells by targeting TLR9 to promote autophagy.

### miR-342-5p Induces Autophagy by Targeting TLR9 to Alleviate the Inflammation in sepsis Cellular Models

**Fig. 7:**
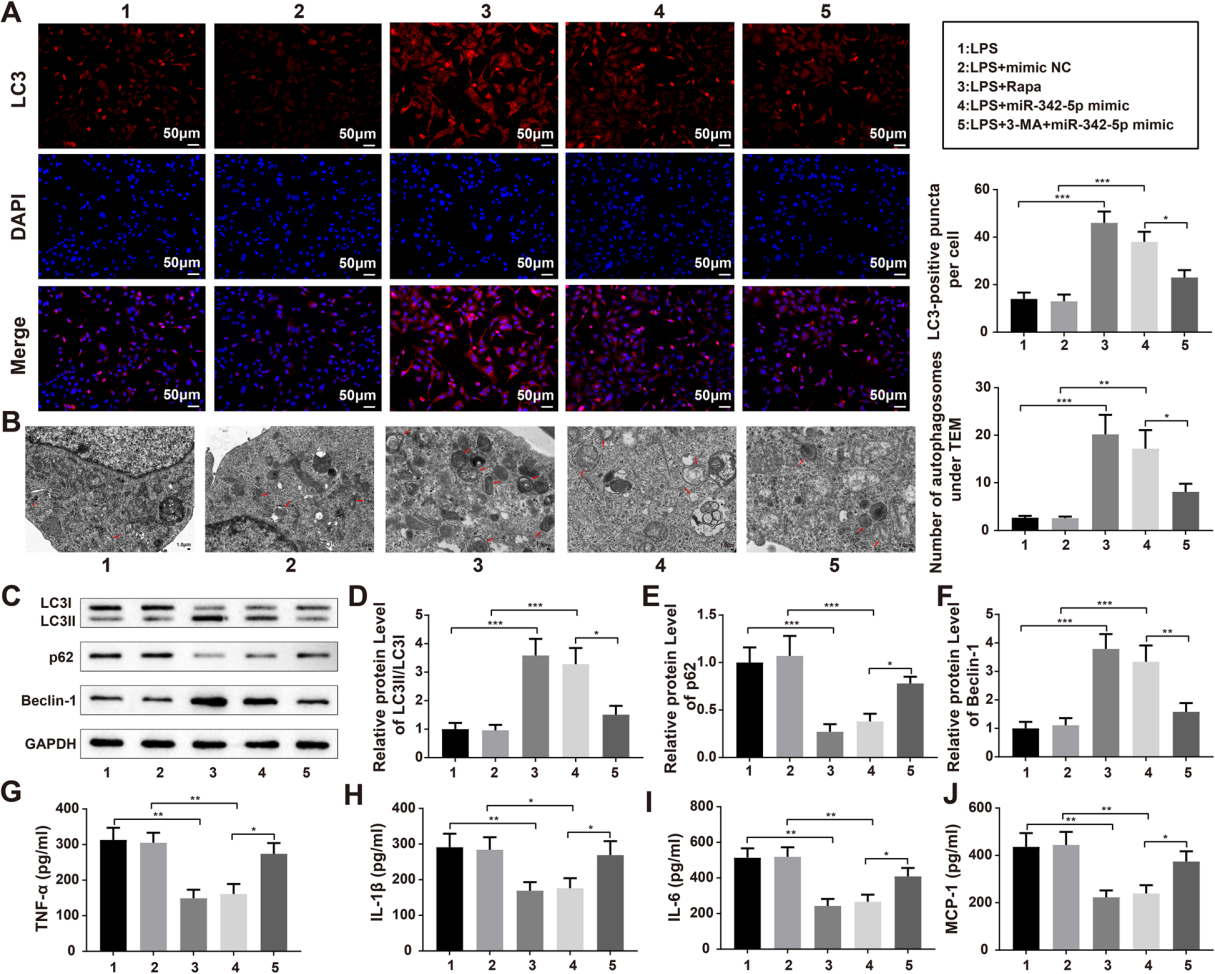
MiR-342-5p alleviates the inflammation of sepsis cell models by promoting cell autophagy. Notes: Following treatment with 10 µM Rapa or 3-MA for 30 min, HK-2 cells were given 1 µg/mL LPS and then transfected with miR-342-5p mimic. The fluorescence intensity of LC3 in LPS-induced HK-2 cells was examined by immunofluorescence staining (**A**). TEM was utilized to observe the number of autophagosomes (**B**). Then, western blot (**C**) analyzed the protein expression levels of autophagy-related proteins LC3-II/LC3-I (**D**), p62 (**E**), and Beclin-1 (**F**). The levels of inflammatory cytokines TNF-α (**G**), IL-1β (**H**), IL-6 (**I**), and MCP-1 (**J**) in the supernatant of LPS-induced HK-2 cells were inspected by ELISA; ^*^ *P* < 0.05, compared to the LPS + mimic NC group or LPS + miR-342-5p mimic group; *N* = 3. ^**^ *P* < 0.01, compared to the LPS group; ^***^ *P* < 0.001, compared to the LPS group or LPS + mimic NC group; lipopolysaccharide; Rapa, Rapamycin; 3-MA, 3-Methyladenine; TEM, transmission electron microscope

HK-2 cells were treated with 10 µM Rapa or 3-MA for 30 min, exposed to 1 µg/mL LPS, and then transfected with the miR-342-5p mimic. The findings obtained from immunofluorescence, TEM, and western blotting showed that treatment with Rapa or miR-342-5p mimic contributed to elevated autophagy in LPS-induced HK-2 cells (Fig. 7A-F, *P* < 0.001, vs. LPS group/LPS + mimic NC group), whereas pretreatment with 3-MA reversed the effect of miR-342-5p mimic on autophagy in sepsis cellular models (Fig. 7A-F, *P* < 0.05 vs. LPS + miR-342-5p mimic group). ELISA demonstrated diminished levels of TNF-α, IL-1β, IL-6, and MCP-1 in the LPS + Rapa group (*P* < 0.01, vs. LPS group) and LPS + miR-342-5p mimic group (*P* < 0.05, vs. LPS + mimic NC group) and enhanced inflammatory cytokine levels in the LPS + 3-MA + miR-342-5p mimic group (Fig. 7G-J, *P* < 0.05, vs. LPS + miR-342-5p mimic group).

**Fig. 8:**
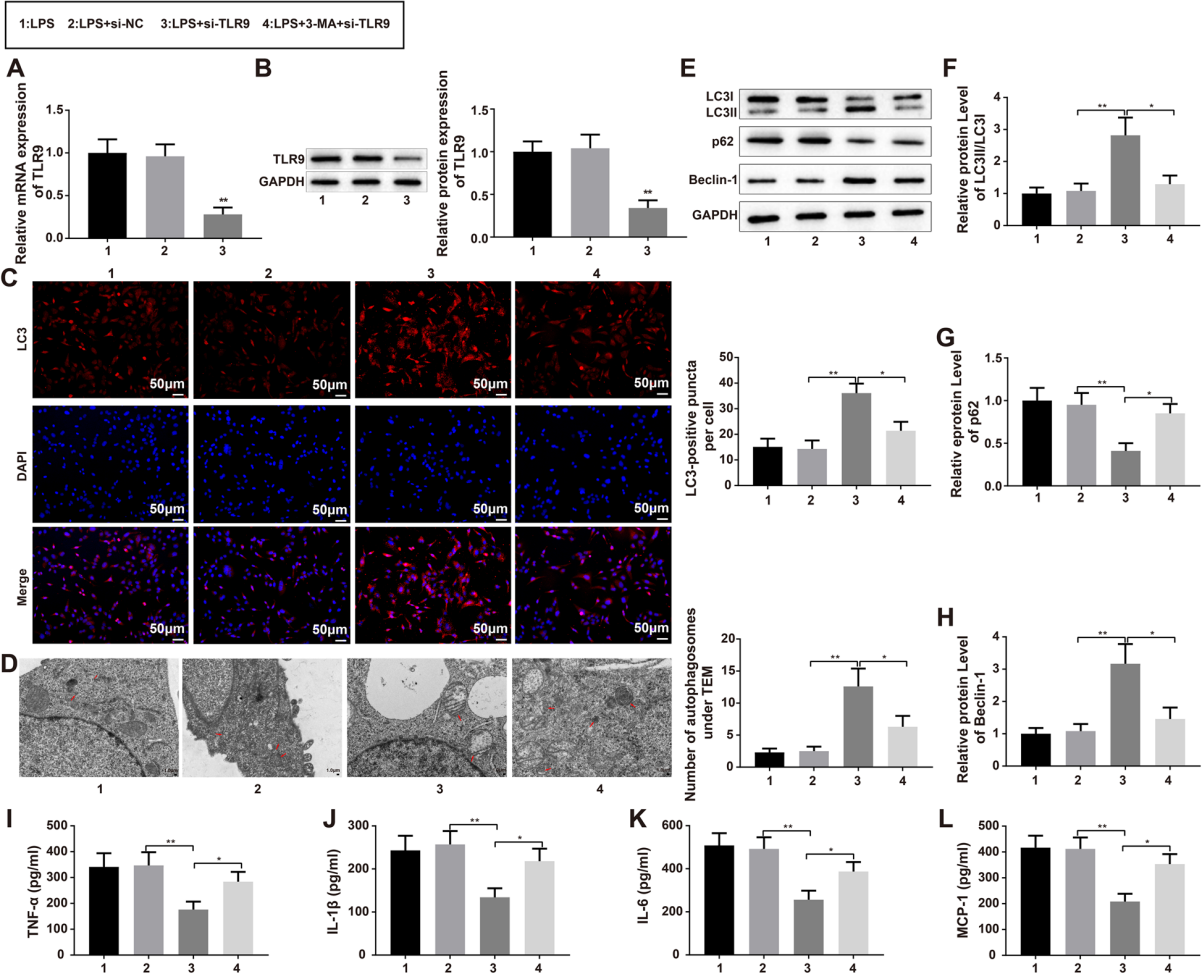
Knockdown of TLR9 promotes cell autophagy to ameliorate LPS-induced inflammation of HK-2 cells. Notes: After sepsis cell models were transfected with si-TLR9, qRT-PCR and western blot were employed to examine the transfection efficiency of si-TLR9 (**A**, **B**). Additionally, the HK-2 cells were subjected to 3-MA for 30 min, exposed to 1 µg/mL LPS and then transfected with si-TLR9. The immunofluorescence was utilized for measurement of fluorescence intensity of LC3 (**C**), TEM for observation of the number of autophagosomes (**D**), and western blot (**E**) for analysis of protein expression levels of autophagy-related proteins LC3-II/LC3-I (**F**), p62 (**G**), and Beclin-1 (**H**). Then, ELISA was applied to detect the contents of inflammatory cytokines TNF-α (**I**), IL-1β (**J**), IL-6 (**K**) and MCP-1 (**L**) in the supernatant of LPS-induced HK-2 cells; ^*^ *P* < 0.05, compared to the LPS + si-TLR9 group; *N* = 3. ^**^ *P* < 0.01, compared to the LPS + si-NC group; lipopolysaccharide; 3-MA, 3-Methyladenine; TEM, transmission electron microscope. Data were analyzed using one-way analysis of variance and Tukey test was used for post hoc analysis. Data were generated from three independent experiments

Subsequently, the cellular sepsis models were transfected with si-TLR9, and qRT-PCR and western blotting were used to determine transfection efficiency. The results demonstrated that TLR9 was successfully downregulated in LPS-treated HK-2 cells (Fig. 8A-B, *P* < 0.01). To investigate the role of TLR9 in the autophagy of sepsis cell models, HK-2 cells were stimulated with 3-MA for 30 min prior to exposure to 1 µg/mL LPS and transfection with si-TLR9. As shown by immunofluorescence, TEM, and western blotting, knockdown of TLR9 enhanced autophagy in LPS-treated cells (Fig. 8C-H, *P* < 0.01), while exposure to 3-MA reversed the effect of si-TLR9 on autophagy (Fig. 8C-H, *P* < 0.05). ELISA results showed that transfection with si-TLR9 remarkably repressed the levels of inflammatory cytokines (Fig. 8I-L, *P* < 0.01), whereas pretreatment with 3-MA increased the levels of inflammatory cytokines (Fig. 8I-L, *P* < 0.05). Taken together, these results showed that miR-342-5p may boost cell autophagy by targeting TLR9 to relieve LPS-induced inflammation in HK-2 cells.

### AMSC-Exo-342 Relieves AKI in mice by Promoting Autophagy

**Fig. 9:**
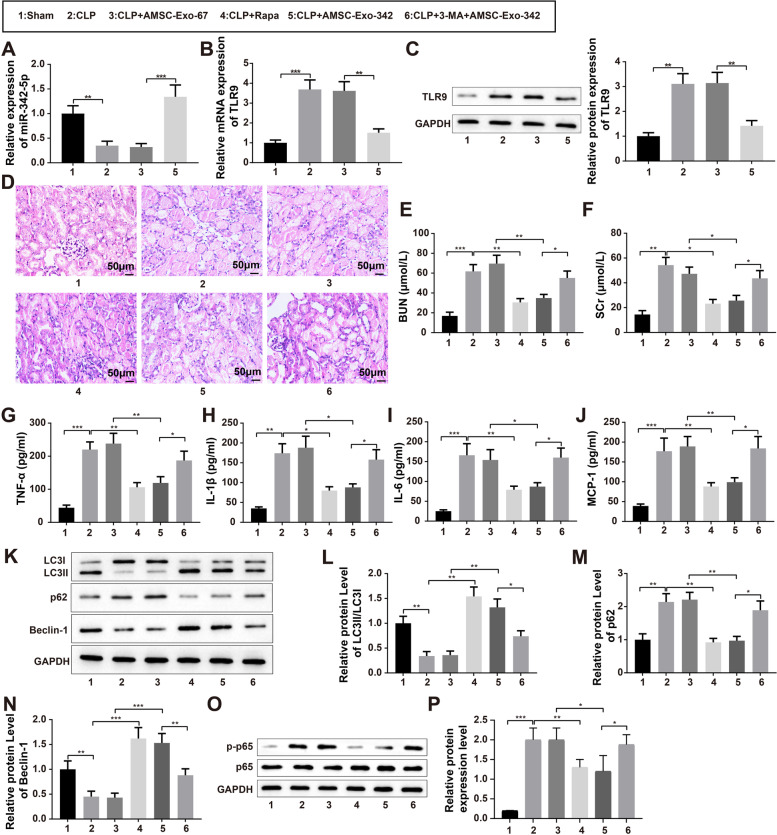
AKI of mice can be relieved by AMSC-Exo-342 through promoting autophagy. Notes: Four hours after establishment of sepsis-associated AKI mouse models by CLP, mouse models were injected with 100 µg AMSC-Exo-342 or AMSC-Exo-67 by the tail vein. Then, 72 h later, the kidney tissues and blood samples were collected. qRT-PCR was applied to measure the expression of miR-342-5p in kidney tissues of mice (**A**), qRT-PCR (**B**) and western blot (**C**) to detect the expression of TLR9 in kidney tissues of mice, and H&E staining to evaluate the damage of kidney (**D**). The levels of BUN (**E**), SCr (**F**), TNF-α (**G**), IL-1β (**H**), IL-6 (**I**), and MCP-1 (**J**) in mouse serum were inspected by ELISA. The protein expression levels of autophagy-related proteins LC3-II/LC3-I (**L**), p62 (**M**), and Beclin-1 (**N**) were determined by western blot (**K**). The expression of p-p65 (**O**, **P**) in kidney tissues was measured by western blot. ^*^ *P* < 0.05, ^**^ *P* < 0.01, ^***^ *P* < 0.001, compared to the Sham, CLP, CLP + AMSC-Exo-67, or CLP + AMSC-Exo-342 group; AMSCs, adipose-derived mesenchymal stem cells; Exo, exosome; AKI, acute kidney injury; CLP, cecal ligation and puncture. Data were analyzed using one-way analysis of variance and Tukey test was used for post hoc analysis. Data were generated from three independent experiments

Sepsis-associated AKI mouse models were established by CLP, in which the mice were injected with 100 µg AMSC-Exo-342 or AMSC-Exo-67 via the tail vein 4 h after CLP. Kidney tissues and blood samples were collected after 72 h. qRT-PCR was used to examine the expression of miR-342-5p in the kidney tissue of mice. The results showed notably increased miR-342-5p levels in the CLP + AMSC-Exo-342 group (*P* < 0.001, vs. CLP + AMSC-Exo-67 group) and suppressed miR-342-5p expression in the CLP group (Fig. 9A, *P* < 0.01, vs. sham group). The findings of qRT-PCR and western blotting highlighted the increase in TLR9 expression in the CLP group (*P* < 0.01, vs. sham group), and the decrease in TLR9 level in the CLP + AMSC-Exo-342 group (Fig. 9B, C, *P* < 0.01, vs. CLP + AMSC-Exo-67 group). Additionally, H&E staining revealed a blurred structure and obvious inflammatory cell infiltration in the kidney tissues and strikingly damaged glomeruli and tubules in the CLP group 72 h after CLP surgery (Fig. 9D). Treatment with AMSC-Exo-342 or Rapa significantly mitigated the damage to the kidney, while intraperitoneal injection of autophagy inhibitor 3-MA into mice reduced the protective effect of AMSC-Exo-342 (Fig. 9D). The results of ELISA showed that CLP surgery increased the levels of serum BUN, SCr, TNF-α, IL-1β, IL-6, and MCP-1 in mice (*P* < 0.01, vs. sham group), and these indices were prominently diminished after treatment with AMSC-Exo-342 or Rapa (Fig. 9E-J, *P* < 0.05, vs. CLP group). However, exposure to 3-MA reversed the effect of AMSC-Exo-342 on the expression of these factors (Fig. 9E-J, *P* < 0.05, vs. CLP + AMSC-Exo-342 group). Western blot analysis of the levels of autophagy-related factors revealed enhanced expression of p62, decreased ratio of LC3-II/LC3-I, and repressed levels of Beclin-1 in the CLP group (*P* < 0.01, vs. sham group) and the CLP + 3-MA + AMSC-Exo-342 group (Fig. 9K-N, *P* < 0.05, vs. CLP + AMSC-Exo-342 group). Additionally, an elevated LC3-II/LC3-I ratio, increased Beclin-1 expression, and decreased p62 expression were observed in the CLP + AMSC-Exo-342 group (*P* < 0.01, vs. CLP + AMSC-Exo-67 group) and CLP + Rapa group (Fig. 9K-N, *P* < 0.01, vs. CLP group). The expression of p-p65 was elevated in kidney tissues of mice in the CLP group but decreased after AMSC-Exo-342 or Rapa treatment (*P* < 0.05) and that further treatment by 3-MA can lead to rebounded expression of p-p65 (Fig. 9O-P, *P* < 0.05). The above results indicated that AMSC-Exo-342 may alleviate AKI in mice by inducing autophagy.

## Discussion

Sepsis is a dominant cause of AKI, and AKI is a frequent complication of sepsis [40]. Recently, the transfer of exosome cargo has been proven to provide a theoretical basis for many stem cell-based treatment outcomes [41]. A previous study confirmed that exosomes obtained from pigment epithelium-derived factor-modified AMSCs are effective in cerebral ischemia–reperfusion injury therapy [42]. In the present study, we found that miR-342-5p functions as a possible molecular cargo of exosomes. Therefore, we speculated that exosomes derived from miR-342-5p-modified AMSCs may alleviate AKI. Then, LPS-induced sepsis cellular models were incubated with exosomes extracted from miR-342-5p-modified AMSCs, and CLP-induced AKI mice were injected with AMSC-Exo-342 to explore the effect of miR-342-5p on AKI as mediated by AMSC-derived exosomes both in vitro and in vivo. We found that AMSC-derived exosomes could deliver miR-342-5p to LPS-induced HK-2 cells to decrease TLR9 expression, which ultimately facilitated cell autophagy, thereby conferring protection against AKI.

Notably, several recent studies have shown that miRNAs can be delivered to recipient cells through an exosome-dependent procedure that alters the gene expression of recipient cells [43, 44]. One of the remarkable findings of this study is the low expression of miR-342-5p in the serum of patients with sepsis-related AKI compared to that in the serum of healthy volunteers. Toward this end, the transfer of miR-342-5p to AKI was explored. Our results revealed that AMSC-derived exosomes may deliver miR-342-5p to HK-2 cells. Additionally, LPS caused elevated levels of TNF-α, IL-1β, IL-6, and MCP-1 in the supernatant of HK-2 cells, showing that there was increased inflammation in our cellular models of sepsis. By exploiting the characteristics of exosome excretion, we showed that the upregulated expression of inflammatory cytokines in HK-2 cells induced by LPS was repressed following the addition of AMSC-secreted exosomes containing miR-342-5p compared with that after exosomes containing the control miR-67. Previous studies have also determined the utility of miR-342-5p, whose expression can be suppressed by ADSC-derived exosomes, in atherosclerosis in human umbilical vein endothelial cell lesion models [45]. Therefore, we confirmed that exosomal transfer of miR-342-5p from AMSCs to LPS-induced HK-2 cells may mitigate inflammation in cellular sepsis models. The effect of AMSC-Exo-342 on LPS-induced HK-2 cells was observed to be disturbed after induced deficiency or upregulation of autophagy. Autophagy mediates intracellular biomass by autodigesting cytoplasmic components [46]. This protective mechanism is activated in cells under stress conditions and can also be aberrantly regulated under certain pathological conditions [47]. During autophagosome formation and autophagy initiation, Beclin-1 binds microtubule-associated LC3-I, which is transformed into its membrane-bound form LC3-II and interacts with p62 [48]. The presence of the LC3-interacting region in p62 is conducive to binding to LC3-II and targeting the cargo of p62 and p62 binding polyubiquitin to the autophagosome for degradation [49]. In this study, autophagy was found to be involved in the pathogenesis of AKI, as evidenced by the repressed autophagy levels in the cellular sepsis models. Furthermore, AMSC-derived exosomes may alleviate LPS-induced inflammation in HK-2 cells by delivering miR-342-5p to HK-2 cells to accelerate cell autophagy. A recent study supporting our findings showed that exosomes derived from mmu_circ_0000623-modified AMSCs may prevent liver fibrosis through the activation of autophagy [50]. Nevertheless, in a previous study, miR-342-5p was found to be decreased in AMSC-derived exosomes, which was partially consistent with the results of our study The same study also showed that miR-342-5p can promote the apoptosis of human umbilical vein endothelial cells through the mitochondrial-dependent apoptotic signaling pathway [45]. This discrepancy may be explained by differences in disease background, which also calls for the validation of the role of miR-342-5p in regulating cell apoptosis and autophagy in different backgrounds.

TLR9 is a cytosolic receptor for unmethylated CpG DNA found in DNA viruses and microbial DNA [51]. A previous study has suggested that selective renal proximal tubular TLR9 activation may intensify ischemic AKI by influencing necrosis, inflammation, and apoptosis [25]. Here, deficiency of TLR9 or overexpression of miR-342-5p was found to enhance autophagy and diminish the release of inflammatory cytokines in LPS-treated sepsis cellular models. The adverse effects of TLR9 and the protective effect of miR-342-5p on LPS-induced cells prompted us to assume that the miR-342-5p/TLR9 axis may be implicated in ameliorating AKI by accelerating cell autophagy. We further discovered that AMSC-Exo-342 alleviates LPS-induced inflammation in HK-2 cells by targeting TLR9 to enhance autophagy. A previous study has shown that AMSC-conditioned medium can attenuate inflammation, mitigate memory deficits, and decrease beta-amyloid formation by regulating TLRs [52]. In our experiments, we injected the miR-342-5p mimic into AKI mice; however, as the therapeutic efficacy was not obvious, we speculate that there may be substantial degradation in mice after injection of the miR-342-5p mimic. Therefore, AMSC-Exo-342 was injected into the mice. The data presented in the in vivo experiments showed that exposure to AMSC-Exo-342 or Rapa relieved CLP surgery-induced injury of glomeruli and tubules, inflammatory cell infiltration, and blurred kidney tissue structure in mice. Additionally, the levels of BUN, SCr, and inflammatory cytokines in the serum of mice with sepsis-associated AKI were diminished, and the autophagy level in the kidney tissues was increased after AMSC-Exo-342 or Rapa treatment. Collectively, AMSC-derived exosomes may deliver miR-342-5p to HK-2 cells to mitigate AKI by inhibiting TLR9 to facilitate autophagy. In addition, we measured the phosphorylated p65 levels in kidney tissues of CLP-treated mice before and after Rapa and AMSC-Exo-342 treatment. The results showed that p-p65 levels were elevated in CLP- treated mice, but decreased in response to Rapa and AMSC-Exo-342 treatment, possibly implicating the NF-κB signaling pathway in miR-342-5p mitigated AKI. It is reasonable to suggest that renal ischemia–reperfusion injury, a major cause of AKI, can be ameliorated by suppressing the activation of the NF-κB signaling pathway and inflammation [53].

Despite these promising results, this study has some limitations that warrant further discussion. For example, autophagy is a complicated process of programmed cell death, which can be triggered and mediated by several mechanisms. This study only focused on the interplay between miR-342-5p and TLR9 in MSC-derived exosomes and autophagy. In this regard, a comprehensive understanding of AKI requires further elaborate studies on inflammation and cell autophagy. Additionally, it is necessary to improve the methods for AMSC culture and exosome purification to increase the feasibility and safety of AMSC-derived exosome therapy in clinical applications.

## Conclusion

In summary, our current study preliminarily reveals a possible mechanism by which miR-342-5p mediated by AMSC-derived exosomes could protect HK-2 cells against inflammation. These findings may provide valuable insights into the role of miR-342-5p in the pathogenesis of AKI and further indicate the potential of the miR-342-5p/TLR9 axis as a novel therapeutic target for AKI.

## Data Availability

The datasets used or analyzed during the current study are available from the corresponding author on reasonable request.
